# Rising Stars: Astrocytes as a Therapeutic Target for ALS Disease

**DOI:** 10.3389/fnins.2020.00824

**Published:** 2020-07-28

**Authors:** Michal Izrael, Shalom Guy Slutsky, Michel Revel

**Affiliations:** ^1^Neurodegenerative Diseases Department at Kadimastem Ltd., Nes-Ziona, Israel; ^2^Department of Molecular Genetics, Weizmann Institute of Science, Rehovot, Israel

**Keywords:** amyotrophic lateral sclerosis, astrocytes, TDP-43 aggregates, astrocyte cell-based therapy, A1 astrocyte, A2 astrocyte

## Abstract

Amyotrophic lateral sclerosis (ALS) is a multifactorial disease, characterized by a progressive loss of motor neurons that eventually leads to paralysis and death. The current ALS-approved drugs modestly change the clinical course of the disease. The mechanism by which motor neurons progressively degenerate remains unclear but entails a non-cell autonomous process. Astrocytes impaired biological functionality were implicated in multiple neurodegenerative diseases, including ALS, frontotemporal dementia (FTD), Parkinson’s disease (PD), and Alzheimer disease (AD). In ALS disease patients, A1 reactive astrocytes were found to play a key role in the pathology of ALS disease and death of motor neurons, via loss or gain of function or acquired toxicity. The contribution of astrocytes to the maintenance of motor neurons by diverse mechanisms makes them a promising therapeutic candidate for the treatment of ALS. Therapeutic approaches targeting at modulating the function of endogenous astrocytes or replacing lost functionality by transplantation of healthy astrocytes, may contribute to the development of therapies which might slow down or even halt the progression ALS diseases. The proposed mechanisms by which astrocytes can potentially ameliorate ALS progression and the status of ALS clinical studies involving astrocytes are discussed.

## Introduction

In Amyotrophic lateral sclerosis (ALS), selective degeneration of both upper and lower motor neurons (MNs) takes place in the central nervous system. Death of MNs leads to rapid and progressive paralysis of target muscles, which causes death within 3–5 years from disease onset, usually due to respiratory failure (Hardiman et al., 2011). The degeneration of MNs is associated with multiple pathophysiological processes including, mitochondrial dysfunction, protein aggregation and formation of inclusion bodies, impairment in RNA processing, elevation in reactive oxygen species (ROS) levels, lack of axonal transport, disruption of the neuromuscular junction and demyelination (Robberecht and Philips, 2013).

The causes for ALS disease are not well understood. The main pathological characteristic of ALS is the accumulation of misfolded proteins and cytoplasmic inclusions in MNs and glial cells, in both motor cortex and spinal cord (Rowland and Shneider, 2001). Around 10–15% of ALS cases are with family history (i.e., familial), and the other cases without family history but still might be genetic (i.e., sporadic) (Kiernan et al., 2011). Familial ALS (fALS) includes mutations of Cu/Zn superoxide dismutase (Rosen, 1993), TAR-DNA-binding protein of 43 kDa (Neumann et al., 2006), fused in sarcoma (Fus) (Kwiatkowski et al., 2009; Vance et al., 2009) and amplification of hexanucleotide (GGGGCC) repeat expansions in the chromosome 9 open reading frame 72 (C9orf72) (DeJesus-Hernandez et al., 2011; Renton et al., 2011). In these mutations, misfolded mutated proteins are spreading (i.e., TDP-43, hSOD1, and FUS), and their aggregation induces severe neuropathology (McAlary et al., 2019). Interestingly, some of these misfolded proteins are not confined to the familial form of the disease where the mutation is known, but also found in sporadic ALS (e.g., TDP-43 inclusions are found in 97% of sALS patients) (Prasad et al., 2019). The mechanism by which ALS mutated proteins become toxic to MNs may share some similarities with prion aggregation and propagation. For example, C9orf72 RNA can be translated into five different dipeptide repeat (DPR) proteins (Mori et al., 2013a,b) that can spread between cells, similar to TDP-43 misfolded protein (Westergard et al., 2016). Toxicity by C9orf72 mutation can also be facilitated by transcription into long repetitive RNA that forms foci of sense or antisense RNA, which segregate RNA Binding Proteins (RBPs) and interfere with their biological activities (Lagier-Tourenne et al., 2012; Fratta et al., 2013; Gendron et al., 2013; Mizielinska et al., 2013; Wen et al., 2017). In addition to ALS, the role of C9orf72 was also identified as the major genetic cause of frontotemporal dementia (FTD) and FTD-ALS (DeJesus-Hernandez et al., 2011; Renton et al., 2011; Vatsavayai et al., 2019). Furthermore, TDP-43 proteinopathy now also constitutes 45% of all FTD molecular pathologies (Arai et al., 2006; Ferrari et al., 2011; Hergesheimer et al., 2019). Misfolded protein inclusions are not restricted to ALS and FTD and were also reported in other neurological diseases such as Parkinson’s disease (Trist et al., 2017, 2018), Alzheimer’s disease (Josephs et al., 2014; Nag et al., 2015), and Huntington disease (Gao et al., 2018).

MNs are the main cells that die in ALS. MNs are substantially large cells with axon extensions that reach far distance locations (i.e., from motor cortex to spinal cord and target muscle) (Ragagnin et al., 2019). The size and function of these cells force them to be more active as compared to other cell types in the nervous system, in terms of cytoskeletal dynamics, energy consumption, RNA metabolism, and proteostasis (Vandoorne et al., 2018). Consequently, MNs are more vulnerable to changes in homeostasis, especially to proteinaceous aggregates (Weishaupt et al., 2016).

The key players in maintaining and supporting MN survival in the central nervous system are astrocytes. Astrocytes are the most common cells in the CNS and have multiple functions. In healthy conditions astrocytes regulate the concentration of different neurotransmitters and ions, supply various metabolites and energy, regulate osmolarity, modulate synaptic activity, secrete neurotrophic and neuroprotective factors, promote neurogenesis (Allen and Eroglu, 2017; Verkhratsky et al., 2017) and remyelination (Fasciani et al., 2018), play a role in immune-modulation (Liddelow and Barres, 2017) and blood-brain barrier formation (Sweeney et al., 2019) as well as in the glymphatic system (Louveau et al., 2017).

## Role and Therapeutic Potential of Astrocytes in ALS

Upon insult, stress, or injury in the CNS, astrocytes enter a reactive state, characterized by changes in their morphology and profile of gene expression. Depending on the signal, astrocytes can transform into reactive A1-type neurotoxic astrocytes, or neuroprotective A2-type astrocytes (Liddelow and Barres, 2015). For example, Neuroinflammatory stimuli, such as LPS, yield A1 reactive astrocytes that promote neurodegeneration and neurotoxicity. Formation of A2 is induced by ischemia, the reactive astrocytes which secrete neurotrophic factors promote neuroprotection and neural repair (Baldwin and Eroglu, 2017; Liddelow and Barres, 2017).

Astrocytes of ALS patients present A1 type characteristics and are actors in the non-autonomous cell disease dogma in ALS (Ilieva et al., 2009). The role of astrocytes in the progression of ALS pathology involves several mechanisms that can result in loss of homeostatic functions or gain of toxic functions. ALS Astrocytes isolated from both sporadic or familial post-mortem ALS patients were found to be toxic to healthy MNs in culture (Haidet-Phillips et al., 2011; Meyer et al., 2014). Toxicity to motor neurons was also demonstrated following coculture of direct conversion of SOD1 or C9orf72 mutated ALS patient’s fibroblasts into induced neuronal progenitor cells (iNSC) and subsequent differentiation into astrocytes (i-astrocytes) (Meyer et al., 2014). This toxicity might be mediated by extracellular vesicles secreted by astrocytes containing miRNA such as miR-494-3p (Varcianna et al., 2019) or proteins such as SOD1, phospho-TDP-43, and FUS (Sproviero et al., 2018). Extracellular vesicles such as exosomes and ectosmes contain a specific composition of proteins, lipids, RNA, and DNA Cells (Gurunathan et al., 2019). Recent study demonstrated that mutated astrocytes derived from C9Orf72-iPSC were toxic to MNs via downregulation of antioxidant proteins secretion, the toxic effects of astrocytes were correlated with the length of astrocyte propagation in culture, consistent with the age-related nature of ALS (Birger et al., 2019). Other study showed that secretion of Tumor Necrosis Factor-Alpha (TNFα) by FUS mutated astrocytes was found to contribute MN-toxicity (Kia et al., 2018). Similar results were obtained with hSOD1G93A primary astrocytes co-cultured with either WT MNs or with MNs from ALS mice (Di Giorgio et al., 2007). The toxic effect on MNs was also demonstrated by the addition of astrocyte-conditioned-medium, indicating that the mechanism involves secretion of soluble molecules by mutated astrocytes (Marchetto et al., 2008).

In contrast, healthy astrocytes protect MNs. Recent study provides evidence for the beneficial role that astrocytes play in protecting MNs in ALS (Smethurst et al., 2020). In this study, the authors first demonstrated that iPSC-derived MNs are more vulnerable to seeded TDP-43 aggregation (extracted from sALS post-mortem spinal-cord) than iPSC-derived astrocytes, indicating a cell-type-specific difference in vulnerability. This observation was further validated by the addition of proteasomal-inhibitors that enhanced the formation and propagation of TDP-43 aggregates. Under these conditions, the presence of seeded TDP-43 aggregation significantly increased MNs cell death, but to a much lesser extent in astrocytes. Next, it was shown that TDP-43 pathology spreads from MNs to astrocytes preferentially but could also be observed spreading from astrocytes to motor neurons. Interestingly, co-culture of healthy iPSC-derived astrocytes protects iPSC-derived MNs that were pre-exposed to TDP-43 aggregates for 3 days, by a significant reduction in TDP-43 aggregates and the apoptotic marker caspase-3 in MNs. This demonstrates that the presence of astrocytes protects MNs from seeded TDP-43 aggregation and its toxicity. Intriguingly, the addition of astrocyte-condition-media alone to iPSC-derived MNs, pre-exposed to TDP-43 aggregates, had similar effects on MN. Lastly, the authors demonstrated that highly purified recombinant TDP-43 oligomers reproduced the observed cell-type-specific toxicity (Smethurst et al., 2020).

Together, the data suggest that healthy astrocytes can protect MNs of ALS patients from a distance, through some secreted product. Among the most studied factors secreted by astrocytes are neurotrophins (Poyhonen et al., 2019). Neurotrophins are a family of proteins that induce the survival (Hempstead, 2006), development, and function of neurons (Reichardt, 2006). This includes brain-derived neurotrophic-factor (BDNF), Nerve-growth-factor (NGF) (Schwartz and Nishiyama, 1994), Vascular-Endothelial-Growth-Factor (VEGF) (Sondell et al., 2000), neurotrophin-3 (NT-3) (Thompson et al., 2014), ciliary-neurotrophic-factor (CNTF) (Thompson et al., 2014), Glial cell-derived neurotrophic factor (GDNF) (Rowitch, 2004) and neurturin (NRTN) (Thompson et al., 2014). Lower concentration of neurtrophins were found in the CSF of ALS patients (Ramamohan et al., 2007; Deepa et al., 2011; Mishra et al., 2016; Shruthi et al., 2017) and its supplementation was found to protect MNs (Storkebaum et al., 2005; Bogaert et al., 2010; Krakora et al., 2013; Shruthi et al., 2017). Astrocytes also release extracellular vesicles (Verkhratsky et al., 2016) that might target near or long-distance sites with a potential selectively to neurons (Venturini et al., 2019). For example astrocyte-derived extracellular vesicles were proven positive for neuroglobin, a protein functioning as neuroprotectant against cell insult (Venturini et al., 2019). Other groups of proteins secreted by astrocytes found to protect neurons are metalloproteases and their inhibitors (Gardner and Ghorpade, 2003) or immune-modulatory factors (Jha et al., 2019).

## Therapeutic Approaches Targeting Astrocytes in ALS

An interesting hypothesis is that in early-stages of ALS disease (pre-symptomatic stage), a period that may take years, there is a process of astrocyte transformation toward the A2-phenotype with neuroprotective properties. This would support the survival of MNs and delay disease onset. Then, upon disease onset and appearance of motor deficiency symptoms, probably after damage to the MN or astrocytes crosses metabolic threshold, the astrocytes acquire the A1 phenotype with neurotoxic properties. Transcriptomic data shows that astroglia in late stage of disease progression in ALS mouse model acquire A1-reactive astrocytic phenotype (Miller et al., 2017). In ALS patients’ reactive astrocytes are observed in susceptible areas and the level of reactivity correlates with the neurodegeneration stage of ALS patients. These astrocytes are convoyed with numerous abnormalities of signaling pathways such as impaired lactate transport (Ferraiuolo et al., 2011), reduction of GLT-1 expression (Martorana et al., 2012), activation of p75-receptor signaling and elevation in pro-inflammatory signaling (Hashioka et al., 2009). In G93A-SOD1 mouse model, reducing mutant SOD1 in astrocytes was found to delay the disease progression, but not disease onset indicating supporting role of astrocytes toward disease onset (Yamanaka et al., 2008).

This hypothesis raises questions of (1) What is the astrocyte profile (A1 vs. A2) at different disease stages? What characterizes the specific threshold that changes the balance between A1 to A2 astrocytes? (2) Can A2 reactive astrocytes transform directly into A1 astrocytes and vice versa? Answers to these questions may provide tools to interfere at specific transformation checkpoints and exploit astrocytes neuroprotective properties to treat ALS. Thus, targeting astrocytes offers a promising approach to treat ALS and maybe also other neurological conditions.

## Astrocyte-Based Cell Therapy

One therapeutic approach is to restore the functionality of endogenous malfunctioning astrocytes by transplantation of healthy human astrocytes. This might become a double-edged sword approach, in which astrocytes provide neurotrophic factors and neuroprotective support through the reduction of misfolded proteins such as TDP-43 to the diseased MNs from one hand, and from other hand might become malfunctioning or even toxic, A1 reactive astrocytes, once they will be introduced to hostile environment ALS patients CNS enriched with aggregations of mis-folded proteins. Comprehensive preclinical studies demonstrated that transplantation of glial-precursor-cells that were generated from iPSCs, or embryonic-stem-cells (ESC), had the potential to delay disease onset and ameliorate clinical symptoms in rodent models of ALS disease (Lepore et al., 2008; Kondo et al., 2014; Izrael et al., 2018) and shown to be safe (Izrael et al., 2018). However, other studies show that astrocytes acquire toxic neuroinflammatory role in response to the cerebrospinal-fluid from ALS patients (Mishra et al., 2016). The transplantation of human glial-restricted progenitors did not result in motor neuron protection or any therapeutic benefits on functional outcome measures (Lepore et al., 2011). Transplantation of genetically modified GDNF neural-stem cells presented efficient delivery of GDNF and preservation of motor neurons, however, MNs survival was not accompanied by continued innervation of muscle end-plates and thus resulted in no improvement in ipsilateral limb use (Suzuki et al., 2007). The difference observed between the therapeutic effect of transplanted cells might reside from difference in the population of astrocytes and their progenitors and should be further investigated. These encouraging results led to the currently ongoing first-in-human phase I/IIa clinical study to evaluate the safety and efficacy of intrathecal transplantation of clinical-grade human astrocytes (AstroRx^®^) derived from human ESC in patients with ALS (ClinicalTrials.gov ID-NCT03482050). The advantage of intrathecal cell injection to cerebrospinal fluid is the distribution of human astrocytes throughout the neural axis, where the cells can reach and exert their effects on both upper and lower MNs. In addition, lumbar puncture is a standard clinical procedure and is generally safe. The mechanisms by which the transplanted astrocytes act still need to be fully elucidated. Most likely the astrocytes act by secreting neuroprotective factors that diffuse to the MNs (Gould and Oppenheim, 2011; Izrael et al., 2018; Thomsen et al., 2018). But many questions remain: Where they attach? Can astrocytes migrate from the CSF into the CNS-parenchyma and reach MNs? Will the astrocytes maintain their A2 characteristics in the hostile CNS environment of ALS or might transform to A1-phenotype further affecting disease course? How long will the transplanted astrocytes survive? Will they transform into the A1 state? The precise mechanisms of action that contribute to the astrocytes’ effects *in vivo* still need to be fully understood, to optimize the potential benefit of these cells.

In another study, neural progenitor cells that were manipulated to overexpress glial cell-line derived neurotrophic factor (GDNF) enhanced the survival of MNs survival and attenuated the progression of the disease phenotype after their injection into the spinal cord (Klein et al., 2005) or motor cortex (Thomsen et al., 2018) of ALS rat model. These encouraging results led to a Phase I/IIa clinical trial (ClinicalTrials.gov ID-NCT02943850) aiming to assess the safety of transplantation of GDNF-producing human astrocyte-precursors into the spinal-cord lumbar segment of ALS patients. Finally, a phase-I/IIa clinical trial (ClinicalTrials.gov ID-NCT02478450) will explore the safety of transplanting Human Glial-Restricted-Progenitor Cells (Q-Cells^®^) into the cervical or lumbar region of the spinal cord in subjects with ALS (Lepore et al., 2011).

In these clinical trials, the cells are transplanted locally into the ventral horn (lumbar or cervical regions) in close vicinity to lower MNs. This might allow the cells to directly exert their therapeutic activity on a specific set of neurons as compared to the intrathecal approach.

## Cell-Based Therapy Using Mesenchymal Stem Cells

Another cell-based therapy approach is the use of mesenchymal-stem-cells (MSC). MSC are adult multipotent-precursors that can be derived from bone-marrow or placenta, with the potential to differentiate into osteocytes, chondrocytes, fibroblasts, and adipocytes (Pittenger et al., 1999). MSC are not natural residence of the CNS but can be induced to secrete some of the neurotrophic factors secreted by astrocytes (Bahat-Stroomza et al., 2009). Recently, single-dose transplantation of autologous MSC that were induced to secrete neurotrophic factors (NurOwn) showed that single combined intramuscular and intrathecal transplantation of MSC-NTF cells demonstrated early promising signs of efficacy and shown to be safe (ClinicalTrials.gov ID NCT02017912) (Berry et al., 2019). These results lead to the ongoing multi-dose phase-III clinical trial in rapidly progressing ALS patients (ClinicalTrials.gov ID-NCT03280056). Preclinical studies in ALS animal model showed that transplantation of MSCs, have the potential to reduce MNs death, prolong animal survival and improved motor performance over sham-injected animals (Suzuki et al., 2008; Boucherie et al., 2009; Uccelli et al., 2012; Marconi et al., 2013). Intrathecal injection of autologous undifferentiated bone-marrow-derived MSCs (NEURONATA-R) to ALS patients (ClinicalTrials.gov ID-NCT01363401) resulted in stabilization of the ALSFRS-R score in all patients over 6 months after first cell injection. ALSo, levels of CSF immunomodulatory cytokines such as IL-10, TGF-β, and IL-6 were increased after MSC injection, this suggest that the effect of MSC treatment on ALS patients might be mediated by an immune response (Oh et al., 2015).

## Other Strategies Targeting Astrocytes Activity

Compounds that can improve endogenous astrocyte functionality are also being tested. For example, a group of compounds that encompass astrocytic glutamate uptake. Excessive activation of glutamate receptor in MNs can result in cell death (Lapucci et al., 2017). Astrocytes uptake glutamate through EAAT2 (GLT-1) transporter, thus, increasing GLT-1 transporter expression in astrocytes may improve the survival of MNs. Compounds such as Class II HDAC inhibitor MC1568 (Lapucci et al., 2017), pyridazine derivative LDN/OSU-0212320 (Kong et al., 2014), b-lactam antibiotics (e.g., ceftriaxone) (Rothstein et al., 2005), neuroimmunophilin ligand (Ganel et al., 2006), and FDA-approved drug Riluzole (Liu et al., 2019) were found to enhance the expression of GLT-1 in astrocytes and delay symptoms of MN decline and in SOD1^*G93A*^ mice.

Compounds aiming at targeting endogenous-astrocytes to reduce oxidative-stress are of great therapeutic potential. Increasing availability of nicotinamide-adenine-dinucleotide (NAD+), an essential redox molecule (Belenky et al., 2007), leads to increased resistance to oxidative-stress and decreased mitochondrial reactive oxygen production (de Picciotto et al., 2016; Harlan et al., 2016). Activation of the transcription factor, erythroid-derived 2, like 2 (Nrf2) in astrocytes confers protection to neurons in culture and *in vivo* (Vargas et al., 2008; Chen et al., 2009). Treatment with nicotinamide-mononucleotide (NMN) or nicotinamide-riboside (NR) increases NAD+ availability in mutant hSOD1-expressing astrocytes, leading to increased resistance to oxidative-stress and reversion of their toxicity toward co-cultured MNs, through SIRT6 activity to Nrf2 activation (Harlan et al., 2019). Edaravon, the second FDA approved drug for ALS, is a free-radical-scavenger of peroxyl-radicals and peroxynitrite, has been shown to inhibit MNs death *in vivo* by reducing oxidative-stress (Ito et al., 2008). In a phase-III clinical trial (ClinicalTrials.gov, NCT01492686) this drug was found safe and demonstrated safety and efficacy in ALSFRS-R (Writing and Edaravone, 2017).

Other mechanism to reduce astrocyte toxicity is by interfering with neuroinflammatory processes taking place in the progression of the disease. Pro-inflammatory cytokines and inflammatory-mediators are also linked to astrocyte-mediated toxicities. For example, an upregulation of interferon-α (IFNα) receptor in astrocytes was found in the spinal-cord of SOD1G93A mice and sALS, and reducing its expression extended survival in SOD1G93A mice (Wang et al., 2011). Additionally, mutant-SOD1 expressing astrocytes secrete IFN-γ, which induces degeneration in motor-neurons *in vitro* (Aebischer et al., 2011). The pro-inflammatory mediator, prostaglandin-D2 (PGD2), has also been linked to motor neuron death in *in vitro* experiments using co-culture of ES-cell derived human MNs and mutant SOD1 astrocytes (Di Giorgio et al., 2008). Transforming growth-factor β (TGF-β) is a multi-functional cytokine involved in many biological functions, including immune homeostasis, neurotrophic response, and microglial development (Butovsky et al., 2014). Alterations of TGF-β signaling have been implicated in ALS due to gene expression profiles (Phatnani et al., 2013). Next, it was demonstrated that astrocyte-derived TGF-β1 is a negative-regulator of the neuroprotective inflammatory response mediated by microglia and T-lymphocytes in ALS mice (Endo et al., 2015). In summary, several treatments have been tested on ALS animals with the aim of inhibiting or reducing the pro-inflammatory action of microglia and astrocytes (Geloso et al., 2017).

An additional way to interfere with endogenous astrocyte function is gene therapy. The challenges using these therapies are crossing blood-brain-barrier (BBB) and specifically targeting endogenous astrocytes cell population. Several clinical studies such as Tofersen (BIIB067) (Miller et al., 2013), miQure (targets down regelation of C9orf72) (Martier et al., 2019a, b), and VM2020 (Sufit et al., 2017) are already testing a gene therapy approach in the CNS. However, the exact mechanism and effect of these therapies on astrocytes and neurons crosstalk and functionality should be further investigated.

## Conclusion and Open Questions

In conclusion, astrocytes play a central role in ALS and other neurodegenerative diseases. Targeting astrocytes functionality using different therapeutic approaches might provide great benefit to ALS patients. Many questions are still left open, such as what defines a different subpopulation of astrocytes and their response to different pathological insults? What is the crosstalk between astrocytes and the immune system? What is the best site in the CNS for astrocyte transplantation? What is the optimal timing for transplantation during the progression of the disease as well as the dose of cells to be transplanted? Is immunosuppression required in the CNS to prevent graft rejection? Step by step, astrocytes become rising stars and show great promise in the treatment of ALS, based on preclinical studies and preliminary results from clinical trials targeting astrocytes in ALS.

## Author Contributions

MI, SS, and MR designed and wrote the review. All authors contributed to the article and approved the submitted version.

## Conflict of Interest

MI, SS, and MR were employed by company Kadimastem Ltd.
